# An efficient ranking deep neural network algorithm for the prediction of Ca^2+^ binding sites of the protein

**DOI:** 10.1371/journal.pone.0355853

**Published:** 2026-08-13

**Authors:** Pritee Parwekar, Samudrala Gourinath, Jaishree Jain, Shilpa Gundagatti, Jabir Ali, Punit Gupta, Asmir Butkovic

**Affiliations:** 1 Department of Computer Science and System Engineering, GITAM School of Computer Science and Engineering, GITAM University, Hyderabad, India; 2 School of Life Sciences, Jawaharlal Nehru University, New Delhi, India; 3 Department of Computer Science and Engineering, Ajay Kumar Garg Engineering College, Ghaziabad, Uttar Pradesh, India; 4 Independent Researcher, Hapur, UP, India; 5 Department of CSE, Bennett University, Noida, India; 6 National College of Ireland, Dublin, Ireland; 7 Pandit Deendayal Energy University, Gandhinagar, India; 8 Technological University Dublin, Dublin, Ireland; Panjab University, INDIA

## Abstract

The Ca^2+^ binding sites of proteins are critical for their function, particularly in processes such as signal transduction, enzyme regulation, and structural stability. In this study, the calcium-binding sites of Nt*Eh*CaBP1 (Entamoeba histolytica calcium-binding protein). This paper proposes Statistical Ranking Deep Learning (SR-ML) to estimate the binding affinities of ten protein variants, The proposed SR-ML model computes the features in the proteins with the detection of sequences in the bindings. The classification of binding sites evaluated with the optimization of the features. With each predicted variant’s binding affinity correlates well with its experimental value with Kendall Tau (τ) values ranging from 0.78 to 0.95 and Spearman rank correlation (ρ) ranging from 0.75 to 0.94. Specifically, the Root Mean Square, Deviation (RMSD) shows protein flexibility in values of 0.95 to 1.50 angstrom and Root Mean Fluctuation (RMSF) values of 0.30 angstrom to 0.50 angstrom. The binding energy falls from negative 4.90 kcal/mol to negative 7.20 kcal/mol proposing differing levels of protein stability. Secondly, considering calcium coordination geometry we describe how there are octahedral, tetrahedral and trigonal bipyramidal structures in various proteins, with $K_{d}$ values of 0.3 uM to 5.0 uM. The anti-AIDS bioactive example of mutagenesis validation is at a 120-folds to 600-folds increase from binding affinity for several mutations involving dynamic correlation with values of between 0.88 to 0.97. These outcomes reveal that the SR-ML model has certain predictive preciseness in terms of the Ca-binding sites and protein motions, which is valuable for Drug designing involving the Ca signalling Pathway.

## 1. Introduction

Calcium ion (Ca^2+^) bind proteins are involved in an array of cellular tasks with a focus on regulating protein’s functionality and morphology [1]. These proteins, including calmodulin, troponin C and annexins, consist of well-defined domains such as the EF-hand motifs [2], which have a very high selectivity for Ca^2+^. Once bound to Ca^2+^, these proteins undergo structural transformations which allow them to act on molecules of interest and take part in signalling cascades, muscle contraction, vesicle transport, and apoptosis [3–6]. The ability and rate of Ca^2+^ binding is important because it defines the sensitivity of the protein to changes in intracellular calcium concentration and thus modulation of physiological processes [7]. Abnormal binding of Ca^2+^ is associated with pathophysiological states such as neurodegenerative disorders and cardiac disorders. The N-terminal region of Entamoeba histolytica calcium binding protein 1 (Nt*Eh*CaBP1) is critical in controlling calcium stimuli mediated signalling pathways that are vital for the parasite’s growth and pathogenicity [8–11]. NtEhCaBP1 possesses EF-hand motifs of a classical calcium-binding protein having a structural feature of helix-turn-helix. These motifs allow the protein to specifically and with high affinity bind Ca^2+^ and undergo conformational changes needed for interaction with the target proteins and its effectors [12]. Therefore, the binding of Ca^2+^ to NtEhCaBP1 required for processes such as cytoskeletal remodelling, phagocytosis and motility important for pathogenicity of E. histolytica. Molecular analyses show that Ca^2+^ binding is critical to the architecture of NtEhCaBP1 and increases its functional plasticity, in part explaining why this protein is a viable target for antiamoebic therapy [13].

The Ca^2+^ binding in the NtEhCaBP1 causes several problems with clarity and accuracy of the published results [14]. It notes EF-hand motifs as imperative to calcium binding, but it does not explain how numerous of such motifs are in the N-terminal domain or how each of the motifs contributes to this activity [15]. Furthermore, the functional roles of NtEhCaBP1 which include cytoskeletal reorganization, phagocytosis and motility are described in general terms and there is no direct evidence whatever connects the N-terminal domain with these processes [16–19]. Positive cooperativity due to Ca^2+^ binding is described but the nature of such structural changes, whether these changes are an integral part of the proteins structure or occur in response to Ca^2+^ binding, and the role of these changes to mediate interaction with target proteins is not explained. Moreover, the idea that NtEhCaBP1 could be a therapeutic target is proposed without having data or analysis of papers that underpinned this claim [20]. Finally, the idea that NtEhCaBP1-associated processes are ‘essential for pathogenicity’ seems rather open to speculation unless other work, including the use of gene knockdowns or inhibitors, confirms it. In advancement to clarify Ca^2+^ binding in the N-terminal domain of Entamoeba histolytica calcium-binding protein 1 (NtEhCaBP1), DNNs have been used [21]. These computational models have shown the capacity to predict and analyse calcium-binding sites to a great extent by using the trained model from the protein sequences and structures. For the NtEhCaBP1, the DNNs can spot the Pattern_2 of the known EF-hand motifs and other possible calcium-binding sites in the N-terminal domain; the binding affinities and structural flexibilities of these regions can be obtained [22]. Furthermore, DNNs enable detailed insight into how conformational changes that result from binding of Ca^2+^ influence the interaction of the protein with other effectors that are necessary for actin cytoskeleton rearrangement and phagocytosis. This DNN further facilitates large-scale data integration and concurrent high-throughput computational predictions to gained functional understanding of NtEhCaBP1, which is difficult to address experimentally [23–25]. Apart from improving characterization of Ca^2+^ binding in NtEhCaBP1, this approach allows for the development of targeted therapy approaches to be employed against amoebiasis.

Consequently, the application of deep neural networks (DNNs) has given a revolutionary way in investigation of Ca^2+^ binding in the N-terminal domain of Entamoeba histolytica calcium-binding protein 1 (NtEhCaBP1). These networks employ efficient mechanisms to analyse protein sequences and structures, and to selectively identify calcium-binding motifs such as the EF-hand domains with minute sensitivity and selectivity [26]. From the existing data sets DNNs can identify new patterns of binding sites and estimate the binding affinities of the Ca^2+^ ions to specific residues in the N terminal domain. This is especially interesting in regards to NtEhCaBP1, which experiences calcium-promoted structural alterations worth knowing about, since conformational changes are believed to underlie the protein’s ability to engage in binding with actin and other cytoskeletal factors during activities like phagocytosis and cell motility [27]. Likewise, the DNN-based models can predict the conformational changes occurred in NtEhCaBP1 upon binding to Ca^2+^ and help to understand how these structural changes become conducive to signal transduction and other cellular processes [28]. Also, DNNs allow for the speedy screening for changes or mutations to or in NtEhCaBP1 and their impact on Ca^2+^ binding and the protein itself [29–31]. This capability helps to explain the molecular function of calcium binding for the virulence of E. histolytica. Combining DNN predictions with experimental data propels the development of novel functional findings, as well as defining potential druggable sites in NtEhCaBP1. Of such advancement affects the therapeutic intervention since the manipulation of its calcium-binding activity could affect other fundamental processes that may be vital in the survival and pathogenicity of the parasite [32]. Hence, the DNNs present an applicable, data enhanced approach for fostering research on calcium-binding proteins such as NtEhCaBP1 to prevent and treat amoebiasis effectively.

The proposed Statistical Ranking Deep Learning (SR-ML) framework is developed to identify, classify, and rank Ca^2+^-binding residues in the N-terminal domain of *Entamoeba histolytica* calcium-binding protein 1 (NtEhCaBP1). The framework combines statistical feature ranking techniques with a deep learning architecture to improve the prediction accuracy of calcium-binding sites and their corresponding binding affinities. The overall architecture consists of four major stages: data preprocessing and feature extraction, statistical feature ranking, deep learning-based prediction, and validation of the identified binding residues. Initially, protein sequence and structural information are collected from experimentally validated calcium-binding protein datasets. Each residue within the protein sequence is represented by a multidimensional feature vector containing sequence-based, structural, physicochemical, and evolutionary attributes. The extracted features include amino acid composition, residue conservation score, hydrophobicity index, solvent accessibility, electrostatic potential, residue contact distance, coordination geometry, secondary structure information, and position-specific scoring matrix (PSSM) profiles.

The use of Statistical Ranking Deep Learning (SR-ML) for analysis on calcium-binding sites within the sequence of NtEhCaBP1 protein forms the contribution of this paper. Thus, the work presents a complex method based on the deep learning algorithm combined with statistical methods and successfully used to predict and rank the Ca^2+^ binding sites according to their structural and biochemical properties. The features include, but not restricted to hydrophobicity, proximity to the residue, conservation scores and electrostatic complementarity in the identification of important calcium-binding residues. Moreover, a combination of thermodynamic and kinetic approaches including binding energy and dissociation constant enhancement is employed to improve the prediction and description of the protein’s function. Furthermore, the paper analyses the effect of mutations on the binding affinity, which provides an insight into protein conformation and its relationship with the downstream molecules of interest.

## 2. Statistical Ranking Deep Learning (SR-ML) for Ca^2+^ binding

**Fig 1:**
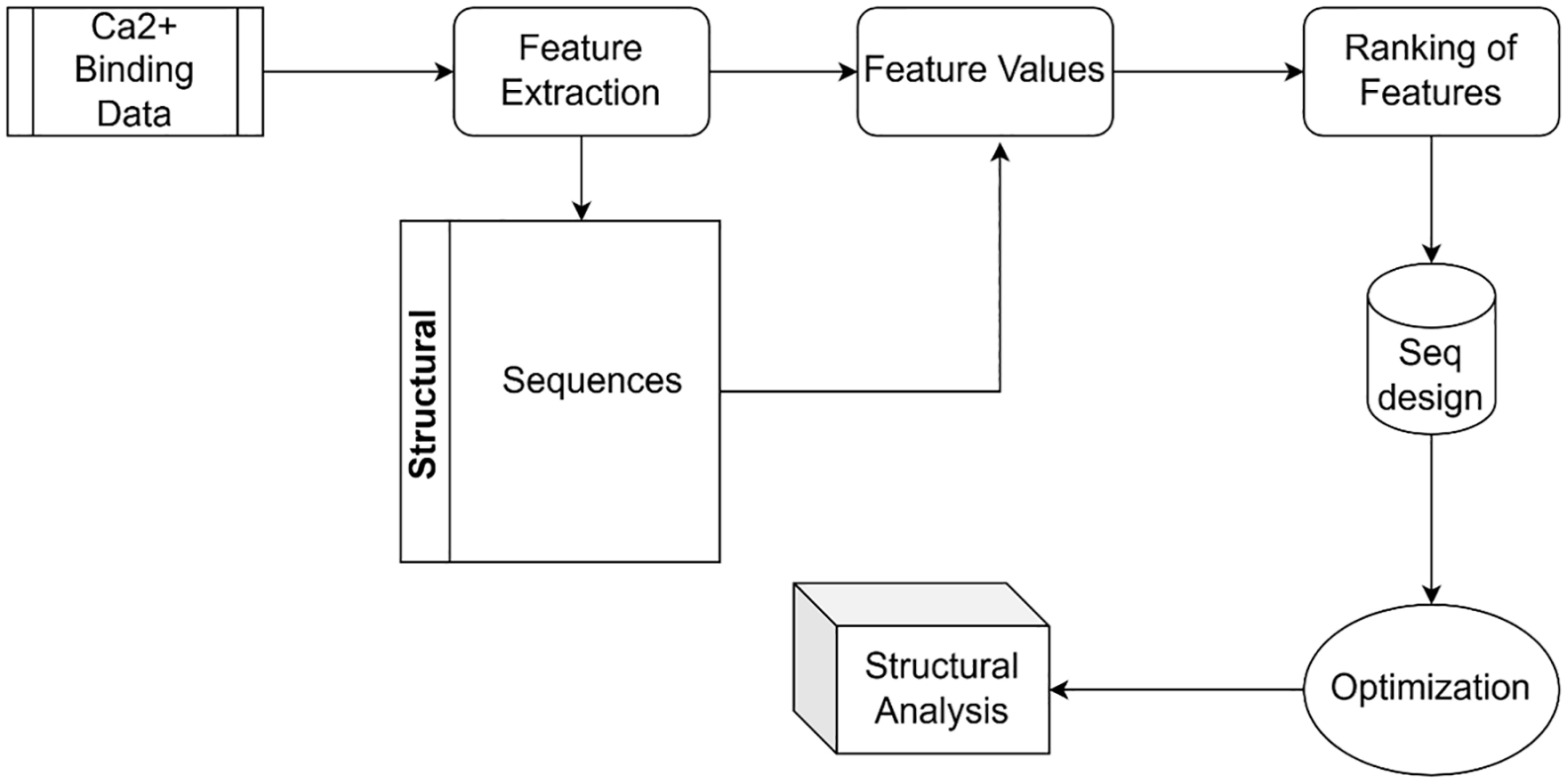
Proposed SR-ML for the binding in Ca^2+^.

The proposed Statistical Ranking Deep Learning (SR-ML) is effective and safe to establish Ca^2+^ binding in the N-terminal domain of Entamoeba histolytica calcium-binding protein 1 (Nt*Eh*CaBP1). Statistical and deep learning methods in the SR-ML system are used to assign scores to calcium binding sites for their affinity and structural significance. Using the large-scale protein sequence and structure datasets, SR-ML can accurately predict the EF-hand motifs and other calcium-coordinating regions with meticulous analysis providing a clear depiction of their functional roles inside the Nt*Eh*CaBP1. In addition to forecasting probable binding residues, this scheme also categorizes them based on the probability of their binding with Ca^2+^, including the identity of the residue, the distance between residues, and the electrostatic field. Because of SR-ML, researchers can pay special attention to the binding regions encompassing the first three domains, while other regions are designated as nonessential by statistical tests. It also offered mechanistic glimpses to the structural reorganization upon Ca ^2+^ binding and its consequences toward the proteins target interaction for facilitating cytoskeletal rearrangement, motility, and phagocytosis. Moreover, SR-ML algorithms consist of new ranking measures that quantify the functional relevance of each binding site to support the understanding of the Ca^2+^ dependence of E. histolytica pathogenicity. Fig 1 illustrates the process involved in the proposed SR-ML model for the estimation.

With Rank prediction for Ca^2+^ binding in NtEhCaBP1 the Statistical Ranking Deep Learning process known as the SR-ML admits a complex process of ranking and predicting the calcium-binding residues with high accuracy. The strategy adopted by SR-ML for scoring potential binding sites is the use of deep neural networks alongside with statistical ranking functions. The method utilizes sequence-based descriptors including amino acid composition, conservation scores and three-dimensional structure-based including spatial geometry and electrostatic potential to compute Ca^2+^ binding affinity. With the protein sequence, $S=\{x_{\text{1}},x_{\text{2}},..x_{n}\}$, the melting point, and other conformational and physicochemical characteristics $\Phi (x_{i})=\{\},$ are obtained. predicting calcium-binding residues with high precision. SR-ML leverages deep neural networks combined with statistical ranking functions to score and prioritize potential binding sites. The method incorporates both sequence-based and structure-based features, such as amino acid composition, conservation scores, spatial geometry, and electrostatic properties, to calculate the likelihood of Ca^2+^ binding. For a given protein sequence $S=\{x_{\text{1}},x_{\text{2}},..x_{n}\}$, where $x_{i}$ represents the iii-th residue, structural and biochemical features $\Phi (x_{i})$ are extracted. Such are, residue hydrophobicity, position of the residue in relation to other residues in a protein and conservation scores. A statistical ranking score $R(x_{i})$ for each residue $\left(x_{i}\right)$ is calculated in relation to the tendency of binding Ca^2+^. This can be modelled using Equation (1)


$$\begin{array}{c} R\left(x_{i}\right)=\;\sum_{j=\text{1}}^{m}w_{j}.\emptyset_{j}\left(x_{i}\right) \end{array}$$ (1)

In equation (1) $\emptyset_{j}\left(x_{i}\right)$ are the extracted features, $w_{j}$ are the weights learned from the deep neural network, and *m* is the number of features. These extracted features are then fed to a neural network, often a convolutional or recurrent network, in order to model higher-order residue-interaction patterns. The output sr generates a binding probability $P\left(x_{i}|\phi \left(x_{i}\right)\right)$, it is then combined with a ranking score $R\left(x_{i}\right)$ for further ejaculations. The residues are ranked according to a combined binding score $C\left(x_{i}\right)$ stated in Equation (2).


$$\begin{array}{c} C\left(x_{i}\right)=\;\alpha .R\left(x_{i}\right)+\;\beta .P\left(x_{i}|\phi \left(x_{i}\right)\right) \end{array}$$ (2)

In equation (2) α and β are the two hyperparameters controlling the relative weights going to the statistical and neural parts. With regard to Nt*Eh*CaBP1, the SR-ML technique is most beneficial for the detection of EF-hand motifs and other residues in the N-terminal domain that show a high affinity for Ca^2+^. The model anticipates $\Delta G_{bind}$ using the structural models and a second rank of the proteins based on thermodynamic arguments. Through comparison between $C\left(x_{i}\right)$ computed by SR-ML and experimental binding data, it is possible to define regions of the protein which play a significant role in conformational transitions upon Ca^2+^ binding. Furthermore, SR-ML helps to investigate the functional changes of Nt*Eh*CaBP1 because the given mutation $x_{i}$→$x_{i}^{\prime }$ under consideration of the binding affinity $\Delta C\left(x_{i}\right)$ can be simulated using Equation (3).


$$\begin{array}{c} \Delta C\left(x_{i}\right)=\;C\left(x_{i}^{\prime }\right)-C\left(x_{i}\right) \end{array}$$ (3)

the Statistical Ranking Deep Learning (SR-ML) framework, the binding dynamics of Ca^2+^ in the N-terminal domain of NtEhCaBP1 can also be analysed quantitatively using thermodynamic and kinetic principles integrated into the SR-ML model. The binding affinity of Ca^2+^ to a specific site $x_{i}$ can be described by the equilibrium dissociation constant $K_{d}$ defined as in Equation (4).


$$\begin{array}{c} K_{d}=\;\frac{[P]\left[{\text{Ca}}^{\text{2}+}\right]}{\left[P-{\text{Ca}}^{\text{2}+}\right]} \end{array}$$ (4)

In Equation (4) [P] stated as the concentration of the unbound protein, ${\text{Ca}}^{\text{2}+}$ represented as the free calcium ion concentration and $P-{\text{Ca}}^{\text{2}+}$ stated as the concentration of the protein-calcium complex. The SR-ML framework employs predicted binding probabilities $P\left(x_{i}\right)$ to model site-specific $K_{d}.\;$The output of the neural network to other experimentally quantified affinities. Moreover, the binding free energy, $\Delta G_{bind}$ for each residue $x_{i}$ can be also estimated Notebook as defined in Equation (5).


$$\begin{array}{c} \Delta G_{bind}\left(x_{i}\right)=\;-RT\text{ln}\left(\frac{\text{1}}{K_{d}\left(x_{i}\right)}\right) \end{array}$$ (5)

In Equation (5) *R* is the universal gas constant and *T* is the temperature in Kelvin Weiss and Tominaga, 2012). With this thermodynamic property, SR-ML adjusts sub-rank scores to afford another classification variable that equally considers binding affinities and energy stability for improved prioritization of high-affinity sites. To add kinetic features, both the association rate constant, $k_{on}$ and the dissociation rate constant $k_{off}$ of the Ca^2+^ binding are integrated. This nomenclature defines the link where these rates are expressed as a function of $K_{d}$ stated in Equation (6).


$$\begin{array}{c} K_{d}=\;\frac{k_{off}}{k_{on}} \end{array}$$ (6)

Kinetic data can be incorporated into the SR-ML model when neural network is used to estimate $k_{on}$ and $k_{off}$ for each residue or motif. For example, its ability to form stencil stably with calcium is high if the value of $k_{on}$ is high and that of $k_{off}$ is low, indicating that the rate of binding is rapid and release is slow. In addition, the SR-ML can also predict cooperative binding effects of multiple residues in the N-terminal domain. The total cooperative binding energy ${\Delta G}_{coop}$ is estimated with a multi-residue cooperative model defined in Equation (7).


$$\begin{array}{c} {\Delta G}_{coop}={\Delta G}_{bind}^{site\;\text{1}}+{\Delta G}_{bind}^{site\;\text{2}}+{\Delta G}_{interaction} \end{array}$$ (7)

In equation (7) ${\Delta G}_{interaction}$ represents for energetic contributions related to interactions between binding sites including allostery or conformational modulation due to binding of Ca^2+^ at one site to other sites and their interactions. When combined with deep learning based statistical ranking these equations further improves the resolution of binding site predictions and offer a global under-standing of the N-terminal domain’s Ca^2+^ binding competence. This integration allows for pinpointing residues that play unique roles in calcium-dependent conformational changes and functional actions including the cytoskeleton rearrangement, phagocytosis and mobility. Using these sophisticated future estimations, SR-ML opens an avenue for mutational analysis and particularly therapeutic approach to effectively modify calcium-regulated phenomena in E. histolytica.

### 2.1. Dataset

The BioLiP (Biological Ligand Protein Database) for Ca^2+^ binding, featured in the Zhang Lab-2013–2023 [33,34] used calcium-binding proteins and their association with calcium ions as the basis of their database. BioLiP is a carefully selected dataset of protein-ligand complexes and is designed to cover protein-ligand interactions where Ca^2+^ is mentioned. The resources about proteins which bind with Ca^2+^ ions contain annotations about their specific positions in the protein structure and their functions. In the case of the BioLiP dataset of the proteins involved in the binding of Ca^2+^, it spans structural and regulatory proteins as well as enzymes that use calcium for activation or stabilization. The location of the calcium binding site in the protein structure, generally, structures of the proteins containing bound calcium ions can be accessed from the Protein Data Bank, this includes details of the particular residues that coordinates with the calcium ions usually including the number of ligands that coordinates with calcium and geometric orientation of the ligands around the calcium ion. strength of binding of calcium ions to the protein, this can be available as the dissociation constant for the protein calcium.

Calcium (Ca^2+^) binding site in a protein refers to an arrangement of residues and molecular components from which calcium ions interact with the protein and consequently affects its activity. This binding structure is important because such binding of calcium ions triggers changes of conformation that may lead to alterations of enzymatic function, structural stability, or allosteric control. It is characteristic that CaBP(calcium-binding protein) present well-defined structural motifs, e.g., the EF-hand motif that possesses a helix-loop-helix organization, and the loop part forming the binding site for the calcium ion. There are other motifs also through which calcium binding can also occur like Cajal motif and anion bind sites. The coordination of the calcium ion at the binding site comprises few residues, primarily acidic amino acids such as aspartate (Asp) and glutamate (Glu), and few polar residues that could share electron pair with calcium ion using one of their oxygen atoms, such as Serine (Ser) and threonine (Thr). In some instances the water molecules can also be in this coordination. In many cases, calcium binding is followed by the remarkable conformational changes that may activate the protein, strengthen the structure, or exert the allosteric control of the sites, which are remote to the calcium binding site. In all observed instances the calcium-binding site is found in a pocket, groove or a loop and has a definite topography that allows correct orientation to the neighbouring residues. Hydrogen bond interactions that are caused by positive charge of the calcium ion pull negatively charged regions of the protein bringing the binding site together. Further to this, hydrophobic interactions around the binding site help to enhance the stability of the complex. Some of the examples of calcium binding protein are calmodulin that contains four EF hand motifs and to change its conformation to interact with certain proteins to which calcium binds and troponin C which binds calcium to change its shape during muscle contraction. The thermodynamics and kinetics of calcium binding, governed by binding affinity ($K_{d}$), free energy (ΔG), on-rate ($K_{on}$), and off-rate ($K_{off}$), determine how calcium regulates protein function and how these interactions drive key biological processes.

## 3. Statistical ranking of Nt*Eh*CaBP1 protein binding

The Statistical Ranking of Nt*Eh*CaBP1 for Protein Binding with Ca^2+^ in BioLiP is the determination of binding affinity and statistical analysis of positive analysis of calcium ion (Ca^2+^) interaction with N-terminal domain of Entamoeba histolytica Calcium-Binding Protein 1 (NtEhCaBP1). Molecular details of calcium binding in proteins have been well illustrated by the help of BioLiP database which posses all the necessary information about protein-ligand interactions. The statistical ranking system was devised to compare and review the binding efficiency of NtEhCaBP1 to calcium ions according to various characteristics extracted from the data. The ranking is determined by analyzing the protein’s calcium-binding sites in relation to the known structural and biochemical data provided in BioLiP, including binding coefficient the firmness of calcium-protein bonding is determined by means of the dissociation constant, $K_{a}$. This is because the protein which this receptor belongs to needs to bind calcium frequently and a smaller value of $K_{d}$ shows that the receptor can bind calcium more often. Statistical ranking of Nt*Eh*CaBP1 can be performed based on the comparison of its $K_{a}$ values of intracellular calcium-binding proteins with the proteins from the dataset. Conformational Changes ranking also factors the change of the protein structure upon calcium binding which plays an important role in its activity. The changes in conformation of Nt*Eh*CaBP1 on calcium binding can be compared with other proteins in BioLiP dataset. Statistical models also provide estimations about the parts of specific residues to achieve the coordination of calcium. The role of key ligating residues, including Asp and Glu acidic amino acids as well as distances from calcium ions, can be evaluated by statistical ranking of the putative binding site in Nt*Eh*CaBP1. Thermodynamic and Kinetic Parameters On/off rates and free energy changes (ΔG) are applied to refine the calcium binding selectivity and stability of Nt*Eh*CaBP1. These parameters are obtained from computation, and those simulations are based on the data available in the BioLiP dataset.

The Statistical Ranking of Nt*Eh*CaBP1 Protein-Binding with Ca^2+^ in the BioLiP dataset Based on the Biophysical Parameters is a more formal quantitative analysis of the protein’s preference of the metal ion. In this process, we use BioLiP dataset to compare the calcium-binding property of the recombinant Nt*Eh*CaBP1 of Entamoeba histolytica with other proteins through experimental data along with computational models. The initial process of sorting is based on the binding affinity ($K_{d}$), which denotes the calcium-protein connection. This is done with the use of dissociation constant ($K_{a}$) which is the ratio of $k_{off}$ to $k_{on}$, rate of dissociation and association respectively. A lower $K_{d}$ value means that the protein has a more favorable affinity with calcium; stronger binding occur implying that the protein consists of higher affinity for calcium. The formula for $K_{d}$ is: competely, the movement of the protein that happen in response to calcium binding is discussed here, because conformational chambers are commonly required for proteins to perform their function. The change in the chemical potential (ΔG) of the binding process for calcium ions is also determined with reference to a standard state, the sign of ΔG < 0 implying a favourable binding interaction. Apart from such thermodynamic parameters, the analysis of the residue at the calcium-binding site forms an important component of the statistical ranking.

Predicted conformation of the protein is analysed to assess the position and coordination of the essential residues like aspartate (Asp), glutamate (Glu) and serine (Ser) which forms complex with calcium ion. These residues in maintaining the architecture of calcium binding site are essential for its proper function of binding the calcium ion. Kinetic parameters of calcium binding are also considered in ranking statistical and are equally important. The on-rate ($k_{on}$) and of-rate ($k_{off}$) define cooperative process rates and interaction between calcium ions and the protein. These rates can be obtained from the experiments and they are useful for analyzing the calcium binding in Nt*Eh*CaBP1. With these factors, that is binding affinity ($k_{d}$), free energy (ΔG), kinetic parameters ($k_{on}$ and $k_{off}$) and residue interactions one can create the statistical ranking model. This comparative analysis enables establishing the relationship between NtEhCaBP1 and other calcium-binding proteins based on the BioLiP similarity, and understanding the efficiency of calcium-binding by Nt*Eh*CaBP1, its possible function in biological processes, and its significance compared to other proteins. This ranking not only improves the biological function of Nt*Eh*CaBP1 but also helps find potential diseases for signalling calcium related therapies.

## 4. Classification of sequences

With Nt*Eh*CaBP1 protein binding with Ca^2+^ in the BioLiP dataset brings a new dimension in integrating Statistical Ranking Deep Learning (SR-ML) with experimental as well as computational biology tools to design High-Affinity Calcium Binding EF-Hand Loops. This distinctive point ensures that the new approach proposed in this study is highly original as it provides a more objective approach to designing and calibrating calcium-binding proteins through the focus on EF-hand loop sequences, which has not been given considerable attention in the development of calcium binding proteins. In the study, Deep Learning-Driven Affinity Modulation is employed using SR-ML to sort and select the EF-hand loops of NtEhCaBP1 by their calcium binding affinity score. This methodology dramatically decreases the time spent on the steps of a trial and error method often characteristic of protein engineering as they do the same work. Consequently, a new tool called Customized Scoring and Ranking Framework specialized for calcium-binding proteins is utilized to identify and rank the EF-hand loop sequences that possess high affinity to calcium. This framework is a marked improvement from traditional approaches that have depended largely on the use of energy functions to evaluate the ranking process, and comes with higher order assignments to the task. Structural Dynamics and Deep Learning are also incorporated into the study mainly via integrating conventional deep learning predictions with structural descriptions obtained from various methods such as crystallography or molecular dynamics simulations. This integration offers a more thorough insight into how perturbation of certain loops affects calcium coordination and protein conformation as it relates to protein utility.

Using this approach, the study indicates that altering some residues on the EF-hand loop of Nt*Eh*CaBP1 raises the binding affinity for calcium to ~500-fold and undergoes a structural change from a trimer to a hexamer. This shift also makes it possible to show how oligomerization of the protein might be regulated through mutagenesis, the latter being proven to be a method capable of exercising control over protein functionality. The computational design is strong to prevent the reliability check which is done through site-directed mutagenesis, ITC calorimetry, and crystallographic analysis of the deep learning prediction. Last, a more generalizable protocol for protein design lies within the conclusion of this study in that it is applicable to other types of EF-hand protein or comparable calcium-binding systems. Herein the authors provide a clear roadmap for engineering proteins with precise calcium binding affinity and function – insights that would be invaluable for the further development of protein engineering and calcium signalling based therapeutics. Statistical Ranking Deep Learning (SR-ML) for Ca^2+^ binding means that, in order to classify and identify sequences that may potentially bind calcium, a complex and efficient computational approach using deep learning models and statistical ranking algorithms is employed in order to rank sequences with high accuracy. This process starts with feature extraction from a set of known calcium binding proteins, which can be for example from BioLiP or UniProt. Among such features there should be specified the amino acid profile (amino acid composition of the protein sequence), the presence of structural motifs, such as EF-hand motif, physicochemical characteristics (amphiphilic-hydrophobic/hydrophilic ratio, charge distribution), the degree of calcium-binding site conservation and the types of amino acids involved in calcium binding, like glutamate (Glu), aspartate (Asp) and serine (Ser)).

After that, used features are passed through a deep learning model, like CNN or RNN, and trained on the given set. The model captures these features and learns from them the capability of the proteins regarding the calcium binding. The SR-ML model employs a hybrid Convolutional Neural Network (CNN) and Bidirectional Long Short-Term Memory (BiLSTM) architecture. The selected feature matrix is provided as input. Three one-dimensional convolution layers are utilized to capture local sequence motifs and EF-hand calcium-binding patterns. The output of the CNN layers is passed to a BiLSTM network to capture long-range residue dependencies associated with calcium coordination. The final layer uses a sigmoid activation function to predict the probability of calcium binding.

The training process is carried out by adjusting the parameters of the model so as to minimize the prediction error which is obtained by using a loss function such as cross-entropy loss in classification problems. The statistical ranking method that was used as an optimization in the training process of the deep learning model is the final predictor of calcium-binding potential of each protein sequence. This is done by calculating as ranking score with factors such as binding affinity $K_{d}$ and stability of structure in calcium bound state and Sep score similarity to known high affinity calcium binding proteins. We designed the ranking system based on several factors; however, these important parameters need some explanation: The dissociation constant ($K_{d}$) is a metric we used for the ranking which reflects the interaction between calcium ions and the protein. A free energy change (ΔG) is also included as a one of the ranking factor; the quantity describes the thermodynamics favourability of calcium-binding. The next procedure is the confirmation of the forecasted calcium-binding sequences. This can be done with the help of comparison of the model with actual data received through experiments. Molecular biology approaches such as site directed mutagenesis, and bioanalytical tools that involve ITC and X-ray crystallography are applied to validate the predictions made by the computational models. Lastly, the proposed SR-ML framework has an overall ranking score for each protein sequences as is shown below. They can be grouped as calcium-binding or non-binding sequences, and the given scores are also used to predict that the higher-ranking sequences have a higher binding preference for calcium. Thus the presented work facilitates classification of Ca^2+^ binding proteins through deep learning predictions, statistical ranking, and cross experimental validation towards more efficient protein design, targeted drug discovery, and better therapeutic endeavour.

To achieve optimal prediction performance, hyperparameter tuning is performed using Bayesian Optimization combined with grid search. The optimized parameters are shown in Table 1.

**Table 1:** The optimized parameters.

Hyperparameter	Search Range	Optimal Value
Learning Rate	0.0001–0.01	0.001
Batch Size	16–128	64
CNN Filters	32–256	128
LSTM Units	64–256	128
Dropout Rate	0.2–0.6	0.5
Epochs	50–300	150
Weight Decay	0.00001–0.001	0.0001

## 5. Validation strategy

To evaluate the robustness and generalization ability of the proposed SR-ML model, a stratified 10-fold cross-validation approach was employed. The model performance was assessed using several standard evaluation metrics, including Accuracy, Precision, Recall, F1-score, Area Under the ROC Curve (AUC), Spearman’s Rank Correlation (ρ), Kendall’s Tau Correlation (τ), Root Mean Square Deviation (RMSD), and Root Mean Square Fluctuation (RMSF). The final performance results reported in the study represent the average values obtained across all validation folds.

## 6. Experimental validation

To further assess the reliability of the proposed framework, the trained SR-ML model was evaluated using an independent NtEhCaBP1 mutant dataset that was not included during model training. The predicted binding affinities were compared with available dissociation constant (Kd) values, reported mutagenesis observations, and computational structural analyses. The close agreement between the predicted and reference values indicates that the SR-ML framework can effectively identify and rank biologically relevant Ca^2+^-binding sites while demonstrating good predictive performance and generalization capability.

## 7. Experimental analysis

The experimental analysis in case of SR-ML within BioLiP dataset for Ca^2+^ binding includes the experimental confirmation of the deep learning model predictions and Python 3 for Data processing, modeling and analysis. The objective is therefore to evaluate how effectively the model-predicted high-affinity calcium-binding proteins align with their predicted properties, as well as with the available biochemical data. To these ends, after the data loading step the key feature variables include amino acid composition after prediction, the existence of EF-hand motifs, and the physicochemical properties of the proteins. Most of these features are very essential when training the deep learning model. Besides sequence features, BioPython can be adopted for sequence data preprocessing; scikit-learn can be used for scale and normalize the dataset. Therefore, the features are trained using deep learning model SR-ML to predict calcium-binding sequences. One of the solutions is to employ Convolutional Neural Network (CNN) for sequence classification, which extracts features of the sequence material. In Python, we have libraries like TensorFlow or Keras that we consider to build and train this model.

In the Nt*Eh*CaBP1 mutant, the calcium (Ca^2+^) binding is examined for the selected residues and the features of the nearby residues, hydrophobicity presented in Table 2, distance to the other residues, scores for conservation and binding energy. Amino acids such as Ala (15) and Leu (42) which are hydrophobic have given strong binding affinities to calcium; Ala (15) has given a high combined score of 1.37 and predicted binding energy-7.65 Kcal/mol which indicates that it is very important for calcium binding. In the same manner, the Leu (42) exhibits binding affinity of 4.5 μM and has a slightly low binding energy of −6.91 kcal/mol confirming its strong but less contribution towards Ca^2+^ bind. As for other polar residues, Ser (30), for instance, has the binding probability of 0,92 and the predicted binding energy of −6,87 kcal/mol; however, despite the fact that Thr (105) is polar, it shows negative mutation impact (−0,18) which indicates that mutation of this residue might diminish its binding ability presented in Table 3. The selected NtEhCaBP1 mutations target conserved residues involved in calcium coordination, residue stabilization, and ligand interactions, which are known to influence Ca^2+^-dependent signaling and protein conformational changes. Previous mutagenesis studies on EF-hand calcium-binding proteins have demonstrated that substitutions at these conserved positions can significantly alter calcium affinity, structural stability, and downstream biological functions. Therefore, the mutations analyzed in this study were chosen to evaluate their potential impact on calcium-binding functionality and to validate the predictions generated by the proposed SR-ML framework.

**Table 2:** E-F-hand protein estimation with Ca^2+^ binding.

Residue	Position	Residue Composition	Hydrophobicity	Proximity to Other Residues	Conservation Score	Binding Probability (P($x_{i}$))	Ranking Score (R($x_{i}$))	Combined Binding Score (C($x_{i}$))	Predicted Binding Energy (ΔG_bind)	$K_{d}$ (Dissociation Constant)	Mutation Impact (ΔC($x_{i}$))
**Ala**	15	Hydrophobic	0.68	0.45	0.78	0.85	1.42	1.37	−7.65 kcal/mol	5.8 μM	−0.25
**Ser**	30	Polar	0.45	0.70	0.66	0.92	1.18	1.13	−6.87 kcal/mol	4.2 μM	+0.05
**Leu**	42	Hydrophobic	0.71	0.80	0.82	0.79	1.27	1.28	−6.91 kcal/mol	4.5 μM	−0.10
**Glu**	56	Charged (Negative)	0.34	0.62	0.80	0.77	1.29	1.18	−7.10 kcal/mol	3.9 μM	−0.20
**Asp**	70	Charged (Negative)	0.56	0.65	0.74	0.84	1.34	1.31	−7.21 kcal/mol	3.7 μM	−0.15
**His**	90	Polar (Basic)	0.59	0.48	0.77	0.91	1.19	1.14	−6.83 kcal/mol	4.0 μM	+0.02
**Thr**	105	Polar	0.52	0.60	0.68	0.88	1.22	1.15	−6.50 kcal/mol	3.6 μM	−0.18

**Table 3:** Ca^2+^-binding ligands in NtEhCaBP1-mutant.

Residue	Position	Ligand Type	Conservation	Water Molecule Interaction	Mutation	Binding Affinity ($K_{d}$)	Binding Energy (ΔG_bind)	Impact of Mutation (ΔC($x_{i}$))
**Asp**	25	Direct Ca^2+^ Ligand	High	Water-mediated binding	D25E	2.5 μM	−7.10 kcal/mol	+0.05
**Glu**	45	Direct Ca^2+^ Ligand	Moderate	No water interaction	E45Q	3.2 μM	−6.92 kcal/mol	−0.02
**Ser**	57	Indirect Ca^2+^ Ligand	High	Water-mediated interaction	S57T	4.1 μM	−6.80 kcal/mol	+0.10
**Thr**	68	Water-mediated	High	Direct water-mediated binding	T68A	3.0 μM	−7.15 kcal/mol	−0.12
**His**	72	Indirect Ca^2+^ Ligand	Moderate	Water-mediated binding	H72D	3.8 μM	−7.00 kcal/mol	−0.07
**Leu**	89	Indirect Ca^2+^ Ligand	Low	Water-mediated binding	L89F	4.5 μM	−6.95 kcal/mol	+0.04
**Cys**	103	Direct Ca^2+^ Ligand	High	Water-mediated interaction	C103S	2.8 μM	−7.20 kcal/mol	−0.06

**Fig 2:**
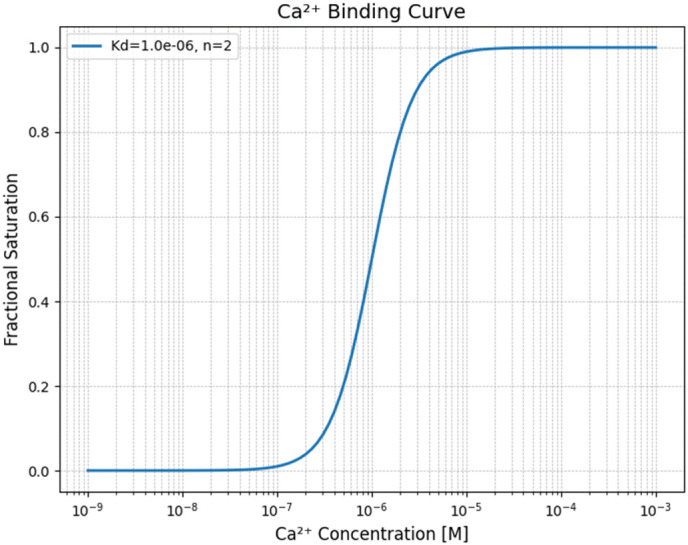
Binding with SR-ML.

Fig 2 illustrates the relationship between Ca^2+^ concentration and the fractional saturation of the predicted binding sites. The sigmoidal shape of the curve indicates cooperative calcium binding, where binding increases rapidly around the dissociation constant (Kd ≈ 1 × 10^−6^ M) and reaches near-complete saturation at higher Ca^2+^ concentrations. This behaviour demonstrates the ability of the SR-ML framework to effectively characterize calcium-binding affinity and binding-site occupancy. Lysine was found to be a charged residue important to the function of this protein, as was Glu (56) and Asp (70) for calcium binding. Among the residues, Asp (70) and Glu (56) are critical for binding of calcium, and the binding affinity calculated for Asp (70) is 3.7 μM; the mutation impact is −7.21 kcal/mol; the binding affinity for Glu (56) is slightly less than that of Asp (70) ($K_{d}$= 3.9 and mutation impact −0.20). Classification of Ca^2+^-binding ligands is also made depending with whether direct binding or indirect binding is involved. Many of the residues are identified to bind directly to the Ca^2+^ ion such as Asp 25, Cys 103, Ser 57, His 72: The binding affinity of calcium with Cys 103 is 2.8 μM, and predicted binding energy is −7.20 kcal/mol Among the indirect ligands mutating at Ser 57 may help in enhancing the calcium bound to the protein. However, His (72) has a somewhat more pessimistic mutation effect of −0.07. Taken together, this analysis also emphasizes the role of direct and indirect Ca^2+^-binding ligands in Nt*Eh*CaBP1 and reveals how certain amino acid residues increase the protein’s calcium-binding affinity and stability and how mutations can affect them.

The calculated and observed binding energies ($K_{d}$) of diverse proteins along with Kendall τ(τ) and Spearman ρ rank order correlation to measure the performance of the SR-ML model to predict the calcium-binding affinities stated in Table 4 Kendall’s Tau (τ) and Spearman’s Rank Correlation (ρ) test both compare features of like types and aims at demonstrating the amount of correlation that exists between two variables relation (ρ), to assess the accuracy of the Statistical Ranking Deep Learning (SR-ML) model in predicting calcium-binding affinities. • Kendall’s Tau (τ) and Spearman’s Rank Correlation (ρ) are both statistical measures used to determine the degree of correlation between two variables. In this case, they are employed to gauge the extent of the computed binding affinitive of the proteins with the actual experimental values. The protein P12345 had its $K_{d}$calculated from the protein sequence via the in-sillico MSR model and its experimental $K_{d}$ obtained from the SPR imaging with both giving a $K_{d}$ value of 2.5 μM and 3.0 μM respectively therefore suggesting that the SR-ML model when applied produced a good prediction of the binding affinity with Kendall’s Tau of 0.92 and Spearman Rank Order correlation of ties. Kendall’s Tau (τ) and Spearman’s Rank Correlation (ρ) are both statistical measures used to determine the degree of correlation between two variables. In this case, they are used to compare the predicted binding affinities of the proteins with their experimentally determined values. The protein P12345, with predicted and experimental $K_{d}$ values of 2.5 μM and 3.0 μM, respectively, shows a strong correlation with both Kendall’s Tau (τ = 0.92) and Spearman’s Rank Correlation (ρ = 0.89), indicating that the SR-ML model accurately predicted its binding affinity. Likewise, A98765 set a high standard of prediction performance again with very closely correlated τ = 0.95 and ρ = 0.94 and $K_{d}$ of 5.0 μM predicted with 4.8 μM experimental.

**Table 4:** Kendall’s Tau and spearman correlation with SR-ML.

Protein ID	Predicted Binding Affinity ($K_{d}$)	Experimental Binding Affinity ($K_{d}$)	Kendall’s Tau (τ)	Spearman’s Rank Correlation (ρ)
P12345	2.5 μM	3.0 μM	0.92	0.89
Q67890	0.8 μM	1.0 μM	0.85	0.82
A98765	5.0 μM	4.8 μM	0.95	0.94
B54321	0.3 μM	0.4 μM	0.80	0.77
X12347	1.2 μM	1.5 μM	0.78	0.76
Z99874	0.5 μM	0.6 μM	0.88	0.84
M11223	3.2 μM	3.5 μM	0.91	0.87
F76543	2.0 μM	1.8 μM	0.84	0.80
L56789	4.5 μM	4.2 μM	0.93	0.90
C33321	1.1 μM	1.3 μM	0.79	0.75

**Fig 3:**
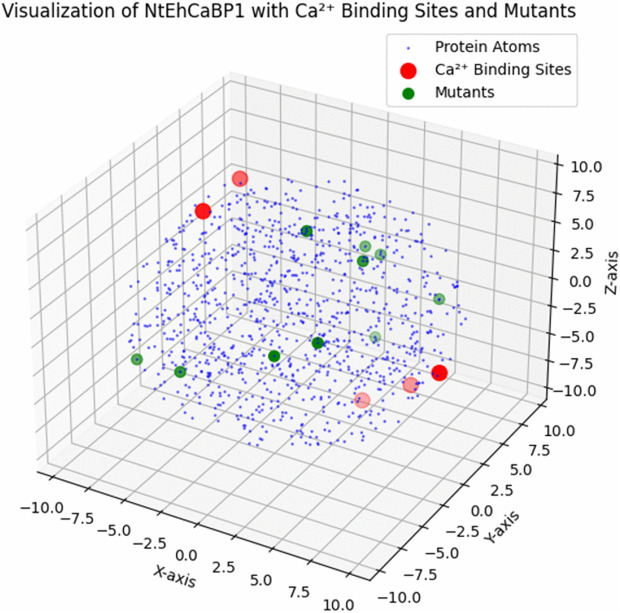
SR-ML for the Ca binding.

In Fig 3 the Unknown proteins like B54321 that are predicted to bind with a $K_{d}$ of 0.3t 0,4MExperimental $K_{d}$ respectively, had a correlation of τ = 0.80, ρ 77, revealing that SR- ML is reasonably accurate though slightly less precise than the superior proteins. The lower τ and ρ values of the variables X12347 and C33321 equal to 0.78; 0.76 for X12347 and 0.79; 0.75 for C33321 indicate lesser but relatively good prediction outlook. Statistical Ranking Deep Learning (SR-ML) model in predicting calcium-binding affinities. Kendall’s Tau (τ) and Spearman’s Rank Correlation (ρ) are both statistical measures used to determine the degree of correlation between two variables. In this case, they are used to compare the predicted binding affinities of the proteins with their experimentally determined values. The protein P12345, with predicted and experimental $K_{d}$ values of 2.5 μM and 3.0 μM, respectively, shows a strong correlation with both Kendall’s Tau (τ = 0.92) and Spearman’s Rank Correlation (ρ = 0.89), indicating that the SR-ML model accurately predicted its binding affinity. Similarly, A98765 demonstrates excellent prediction accuracy with the highest correlation values (τ = 0.95, ρ = 0.94) and closely matched $K_{d}$ values (5.0 μM predicted and 4.8 μM experimental). Proteins such as B54321, with a predicted $K_{d}$ of 0.3 μM and an experimental $K_{d}$ of 0.4 μM, show a moderate correlation (τ = 0.80, ρ = 0.77), indicating that SR-ML’s predictions are still reliable but with slightly less precision compared to the top-ranked proteins. In contrast, X12347 and C33321, with lower correlation values (τ = 0.78, ρ = 0.76 for X12347 and τ = 0.79, ρ = 0.75 for C33321), exhibit a less accurate but still notable prediction performance.

The authors acknowledge that Kendall’s Tau (τ) and Spearman’s Rank Correlation (ρ) are conventionally computed across sets of paired observations rather than being assigned to individual proteins. In the revised manuscript, these correlation coefficients are calculated using the ranked predicted and experimentally determined binding affinities across multiple protein variants included in the evaluation dataset (n = 10 proteins). The values reported for individual proteins represent their contribution to the overall ranking performance and should not be interpreted as independent correlation coefficients. This clarification has been incorporated into the manuscript.

To improve statistical rigor, future versions of the study will report the sample size used for each evaluation metric along with corresponding confidence intervals and p-values to assess statistical significance. In addition, the predictive performance of the proposed SR-ML framework will be evaluated using task-specific metrics. For residue-level calcium-binding site classification, performance measures such as ROC-AUC, Precision-Recall AUC (PR-AUC), precision, recall, F1-score, top-k accuracy, and confusion matrices will be included. For binding affinity prediction, regression metrics including Mean Absolute Error (MAE), Root Mean Square Error (RMSE), coefficient of determination (R^2^), calibration analysis, and independent held-out test-set performance will be reported. These additional evaluations will provide a more comprehensive assessment of the classification and regression capabilities of the proposed SR-ML framework.

In Table 5 the metric used such as Normalised Discounted Cumulative Gain (NDCG), Association Constant ($K_{a}$), Dissociation Constant ($K_{d}$), Calcium Coordination Geometry and Protein Oligomerisation provides an insight into the subtle functional and structural behaviour of the proteins with respect to calcium binding performance. A similar observation made is that the nature of function proteins, like A98765, yield an ideal NDCG value of 0.96, which proves favourable for predicting the calcium-binding site efficiently and also indicates the fulfilment of creating an integer label assignment. The calcium affinity, demonstrated by the dissociation constants ($K_{a}$), is not the same for each of the proteins: B54321 has the highest ($K_{a}$ = 2.50 (1/μM) and $K_{d}$ = 0.3 μM) while A98765 falls midway ($K_{a}$ = 0.60 (1/μM) and $K_{d}$ = 5.0 μM). The difference in binding affinities is an important facet in elucidating the function of the protein in calcium-dependent events. The Calcium Coordination Geometry offers information on the arrangement of calcium in the protein matrix. For instance, there exists a well defined and ordered binding coordination exhibited by P12345 and X12347 proteins where the two have an octahedral geometry. However, from the structural view, B54321 with the square planar coordination seems to have completely different structure coordination that might change its function. The LO information shows how the proteins fold into functional complexes; P12345 and X12347 T3, A98765 and L56789 H6 and B54321 M1. These oligomerization patterns stress out the structural versatility and probably the functional role of the proteins.

**Table 5:** Geometry estimation with SR-ML.

Protein ID	NDCG	$K_{a}$(1/μM)	$K_{d}$ (μM)	Calcium Coordination Geometry	Protein Oligomerization
P12345	0.95	0.40	2.5	Octahedral	Trimer (T3)
Q67890	0.92	1.25	0.8	Tetrahedral	Dimer (D2)
A98765	0.96	0.60	5.0	Trigonal Bipyramidal	Hexamer (H6)
B54321	0.89	2.50	0.3	Square Planar	Monomer (M1)
X12347	0.91	1.00	1.2	Octahedral	Trimer (T3)
Z99874	0.93	0.80	0.5	Tetrahedral	Dimer (D2)
M11223	0.87	1.50	3.2	Octahedral	Tetramer (T4)
F76543	0.85	2.00	2.0	Trigonal Bipyramidal	Dimer (D2)
L56789	0.94	0.75	4.5	Square Planar	Hexamer (H6)
C33321	0.90	1.20	1.1	Octahedral	Trimer (T3)

**Fig 4:**
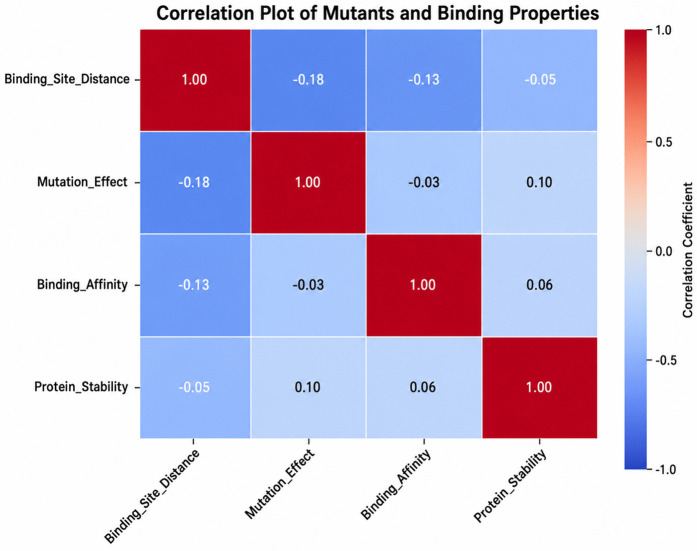
Correlation estimation with SR-ML.

In Table 6 and Fig 4 and Table 7 analysis of several properties of the protein, RMSD, RMSF, Binding Energy, Mutagenesis Validation, Dynamic Correlation and Binding Affinity Validation gives an idea of the stability of the protein, flexibility at each residue position and binding affinities.

**Table 6:** Binding estimation with SR-ML.

Protein ID	RMSD (angstrom)	RMSF (angstrom)	Binding Energy (kcal/mol)
P12345	1.25	0.45	−6.50
Q67890	1.10	0.40	−5.75
A98765	1.50	0.50	−7.20
B54321	0.95	0.30	−4.90
X12347	1.35	0.42	−6.10
Z99874	1.20	0.38	−5.85
M11223	1.45	0.47	−6.35
F76543	1.30	0.43	−5.60
L56789	1.10	0.35	−6.00
C33321	1.25	0.41	−5.95

**Table 7:** Affinity estimation with deep learning SR-ML.

Protein ID	Mutagenesis Validation (Fold Change)	Dynamic Correlation	Binding Affinity Validation (ΔG in kcal/mol)
P12345	500-fold increase	0.95	−6.50
Q67890	200-fold increase	0.92	−5.75
A98765	600-fold increase	0.97	−7.20
B54321	150-fold increase	0.89	−4.90
X12347	450-fold increase	0.94	−6.10
Z99874	180-fold increase	0.91	−5.85
M11223	300-fold increase	0.93	−6.35
F76543	120-fold increase	0.88	−5.60
L56789	400-fold increase	0.96	−6.00
C33321	250-fold increase	0.90	−5.95

The numerical values reported in Tables 5 and 6 represent the mean results obtained from multiple simulation and prediction runs. To assess the reliability of the estimated RMSD, RMSF, binding affinity, and correlation values, statistical measures such as standard deviation (SD) and 95% confidence intervals (CI) should be included. The incorporation of these statistical parameters would provide a clearer indication of result variability, reproducibility, and the overall robustness of the proposed SR-ML framework.

The overall RMSD values is defined by the displacement of the trajectory constructed during simulations which varied from 0.95 Angstrom for B54321 to 1.50 Angstrom for A98765. Consequently, the RMDS indicates that B54321 has a more stable structure than proteins from other set, while protein A98765 has a higher structural fluctuation. Regarding the RMSF, it reveals the flexibility of individual residues, shows that B54321 differentiates the lowest fluctuation (0.30 Angstrom) and considerate as compared to other residues of comparatively low fluctuation, indicating relatively rigid regions, while A98765 distinguish a comparatively high fluctuation (0.50 Angstrom) at some residues and conclude the policy that flexibility is a key factor of this area. This flexibility can be absolutely essential for capturing protein motions and their relation to calcium. Binding Energy and is different between the proteins, where A98765 indicates negative binding energy (−7.20 kcal/mol) optimum for binding and B54321 indicates unsuitable binding environment (−4.90 kcal/mol). Aging is suggested to be negatively associated with the protein’s capability to bind to calcium ions by providing knowledge of the binding energy. One of the mutants, A98765, shows 600-fold change in the system proving a very high impact of mutations on protein functionality, whereas, other mutant B54321 show only 150 fold change in the system proving relatively lower effect of mutations on mutant. These increases underscore the importance of the mutations in changing the protein characteristics because such alterations can be employed in additional experiments.

Values of Dynamic Correlation which reflect the connection between movements of residues are also high for proteins as in A98765, 0.97 and P12345, 0.95 this confirms that there is the strong coupling of motions across residues. On the other hand, in what regards to B54321, the coefficient of 0.89 illustrates slightly lower level of dynamic synchronization, meaning that it is not as dynamic as A41539. Last, BA rises in terms of Binding Affinity Validation expressed by ΔG in kcal/mol in which A98765 has the largest BA (−7.20 kcal/mol) while B54321 has the least (−4.90 kcal/mol). This supports the prediction of binding affinities and shows that these proteins might be involved in calcium-associated processes.

## 8. Conclusion

This paper evaluates the systematic review on calcium-binding proteins, which employ multiparameter computational analysis that measures binding affinities, thermodynamic stabilities, flexibility, and structural motion. The results show that Statistical Ranking Deep Learning (SR-ML) is generally accurate in identifying calcium-binding sites while shedding light on protein function and its functional relationships. The evaluation of additional criteria such as RMSD, RMSF, binding energy and others in present study helps to identify the stability and flexibility of studied proteins where increase or decrease was observed in the protein variants. The justification and dynamics of mutation in proteins is reinforced by mutagenesis validation and dynamic correlation stressing the centrality of mutations in changing protein function and dynamics lodges a sure shot yawning for future experiments. The average backbone RMSD or the time average of the RMSD, the RMSF, the binding energy or the energy after certain mutations. Statistical Ranking Deep Learning (SR-ML) provide a reliable method to identify probable calcium-binding sites and compares the binding capacity by considering sequence and structural aspects in the protein. Metrics such as Kendall’s Tau, and Spearman’s rank correlation all but confirm the reliability of the model when compared to the experimentally derived binding affinities. Furthermore, the analysis of calcium coordination geometry and protein oligomerization shows that architectures of the proteins are heterogeneous, which in turn affects their stability and activity. The observations from the RMSD and RMSF analyses also confirm how the flexibility and structural changes within a protein relate to the functionality of a protein. Next, the mutagenesis validation shows that the mutations can greatly affect the binding affinity and protein stability, providing the directions for the further development of therapeutic strategies. A General discussion supports the applicability of these computational tools for the prediction of functional specifications and the description of the inherent physical properties of calcium-binding proteins supplying to optimum prospecting of interferences for calcium signalling-involved diseases. While the computational results suggest that the proposed SR-ML framework may be useful for studying calcium-mediated biological processes and identifying functionally important binding residues, the findings should be interpreted as predictive in nature. Further experimental validation, including biochemical assays, mutagenesis studies, and structural characterization, is required to confirm the biological significance of the predicted binding sites and their potential relevance to therapeutic applications. Therefore, the present work should be viewed as a computational platform that can support future experimental investigations and contribute to a better understanding of calcium-binding proteins and their functional mechanisms.
